# Pathogenesis analysis of pituitary adenoma based on gene expression profiling

**DOI:** 10.3892/ol.2014.2613

**Published:** 2014-10-13

**Authors:** WEIMIN WANG, ZHIMING XU, LI FU, WEI LIU, XINGANG LI

**Affiliations:** 1 School of Medicine, Shandong University, Jinan, Shandong 250012, P.R. China; 2 Department of Neurosurgery, Qingdao Municipal Hospital, Qingdao, Shandong 266071, P.R. China; 3 Department of General Surgery, Qingdao Municipal Hospital, Qingdao, Shandong 266071, P.R. China; 4 Department of Neurosurgery, Qilu Hospital of Shandong University, Jinan, Shandong 250012, P.R. China

**Keywords:** pituitary adenoma, differentially-expressed genes, interleukin 6, signal transducer and activator of transcription 3, POU1F1

## Abstract

The aim of the current study was to investigate the pathogenesis of pituitary adenoma through screening of the differentially-expressed genes (DEGs) and proteins in normal pituitary and pituitary adenoma tissues, and analyzing the interactions among them. Following the acquisition of gene expression profiling data from a public functional genomics data repository, Gene Expression Omnibus, DEGs were screened in normal pituitary and pituitary adenoma tissues. Upregulated and downregulated DEGs were further identified through gene ontology functional enrichment analysis. Subsequently, the DEGs were mapped to the Search Tool for the Retrieval of Interacting Genes database, and the protein-protein interaction (PPI) networks of the upregulated and downregulated DEGs were constructed. Finally, the functional modules of the PPI network of the downregulated DEGs were analyzed. In total, 211 upregulated and 413 downregulated DEGs were screened between the normal pituitary and pituitary adenoma samples. Downregulated DEGs were associated with certain functions, including the immune response, hormone regulation and cell proliferation. Upregulated genes were associated with cation transport functions. Five modules were acquired from the PPI network of the downregulated DEGs. Transcription factors, including signal transducer and activator of transcription 3 (STAT3), interleukin 6 (IL-6), B-cell lymphoma 6 protein, early growth response 1, POU1F1, jun B proto-oncogene and FOS were the core nodes in the functional modules. In summary, the DEGs and proteins were identified through screening gene expression profiling and PPI networks. The results of the present study indicated that low expression levels of hormone- and immune-related genes facilitated the occurrence of pituitary adenoma. Low expression levels of IL-6 and STAT3 were significant in the dysimmunity of pituitary adenoma. Furthermore, the low expression level of POU1F1 contributed to the reduction in pituitary hormone secretion.

## Introduction

The pituitary gland, as an important endocrine organ, performs vital roles in organismal modulation through secreting several key hormones, including prolactin (PRL), growth hormone (GH), adrenocorticotropic hormone and thyroid-stimulating hormone (TSH) (1,2). By regulating the hormone secretion of a target gland, the anterior pituitary participates in the development and normalization of tissues (3). The abnormality of the pituitary may severely disrupt the metabolic homeostasis, damaging various organs to different extents (4). Pituitary adenoma is a distinctive intracranial tumor developing in the anterior pituitary gland, which exhibits the characteristics of a tumor and an endocrine disorder. Pituitary adenoma, which is caused by mutations in a series of significant pituitary genes, including protein kinase C, p16, and growth arrest and DNA damage-45G (GADD45G), has provoked continuous attention among endocrinologists due to its variability in clinical presentation and the unpredictability of tumor growth (5).

As demonstrated in previous studies, pituitary adenoma may affect human development in a negative manner from a variety of perspectives. Excess hormone secretion by the pituitary gland, caused by pituitary adenoma, produces several metabolic disorders and visceral injuries (6). By contrast, other hormones are downregulated due to the compression of the tumor, which subsequently results in the functional decline of the target glands (7).

To date, a wealth of studies have been conducted on the surgical treatment and chemotherapy of pituitary adenoma (8,9). Studies have shown that the gene and protein expression of certain regulatory factors have vital roles in pituitary adenoma. Vogelstein *et al* (10) found that p53 inhibits the development of a pituitary adenoma and this function may be restrained by pleomorphic adenoma gene-like 1 in combination with reprimo, TP53-dependent G2 arrest mediator candidate, p21 and phorbol-12-myristate-13-acetate-induced protein 1. It is well acknowledged that the overexpression of GADD45β may inhibit tumor growth through activating apoptosis-inhibiting factors, which indicates that GADD45β may also serve a potential inhibitor of pituitary adenoma (11). Pituitary adenoma may be associated with an increase or decrease in a variety of gene expression levels; the majority of these changes also exert regulatory effects on tumorigenesis. Although a number of studies have reported controversial effects of pituitary adenoma on the potential target genes, no effective detection method is available using the flux way to systematically detect the gene and protein differential expression caused by pituitary adenoma (12–14).

The present study aimed to investigate the types and changes of gene expression in pituitary adenoma compared with normal pituitary tissues through gene expression profiling. Subsequently, by establishing a protein-protein interaction (PPI) network of differentially-expressed genes (DEGs), the effects of differential proteins on pituitary adenoma, and the interactions among diverse differential proteins were analyzed.

## Materials and methods

### Data preprocessing and acquisition of gene expression profiling

The gene expression profile GSE26966 (11) was downloaded from the public functional genomics data repository, Gene Expression Omnibus (http://www.ncbi.nlm.nih.gov/geo/) database. Among the total 23 samples that were investigated, nine samples were from normal pituitary tissues and 14 samples were pituitary adenoma tissues. The annotation information for all probe sets was provided by Affymetrix Human Genome U133 Plus 2.0 Array (Affymetrix, Inc., Santa Clara, CA, USA). For data processing and differential expression analysis, the probe-level data was converted from the CEL file format into the expression values of a probe matrix by robust multi-array average (RMA) in Affy package (15), and the serial numbers were transferred into gene names by platform R/Bioconductor note package (16). Finally, as one gene has numerous corresponding probes, the average value of all expression value of probes was calculated as the expression value of a single gene.

### DEG screening

The Bayesian linear model of the limma package in the R software (5) was used to identify DEGs in pituitary adenoma tissues compared with those in the normal pituitary gland. Only genes with a log fold-change value of >1.5 and a false discovery rate (FDR) of <0.05 were selected as DEGs. To ensure that the screened DEGs could well characterize the samples, clustering analysis and dendrograms for DEGs were established.

### Functional enrichment analysis of DEGs

Gene ontology (GO) functional enrichment analysis was conducted using the Database for Annotation Visualization and Integrated Discovery (DAVID) online tools (17) to study the functions of upregulated and downregulated DEGs, respectively. The cut-off criteria were the FDR and Bonferroni correction (18); P<0.05 was considered to indicate a statistically significant difference.

### PPI network construction

The PPI pairs were acquired by directly mapping the DEGs to the Search Tool for the Retrieval of Interacting Genes (STRING) database (19); the PPI network was constructed by PPI pairs whose protein interaction scores were >0.7. The protein interaction scores were calculated using the following formula (20):

$$\begin{array}{l} S(\text{e}(x,y))=f(diff(x),corr(x,y),diff(y))\\ =-2\sum_{i=1}^{k}{\text{log}}_{e}(p_{i}) \end{array}$$

Diff(x) and diff(y) represent differential expression assessments of genes x and y, respectively. Corr(x,y) represents their correlation between genes x and y. Where k=3, p_1_ and p_2_ are the P-values of differential expression of two nodes, p_3_ is the P-value (20) of their coexpression.

Specific transcription factors in the PPI network of pituitary adenoma were excavated using the TRANSFAC database (21).

### Analysis of PPI modules

Modules in the PPI network of downregulated DEGs were selected using the cluster ONE package in the Cytoscape software (22). Modules with P-values of network minimum density >0.05 and node numbers >10 were screened. Sequentially, GO analysis of these modules was conducted using DAVID.

## Results

### DEGs in pituitary adenomas

**Figure 1:**
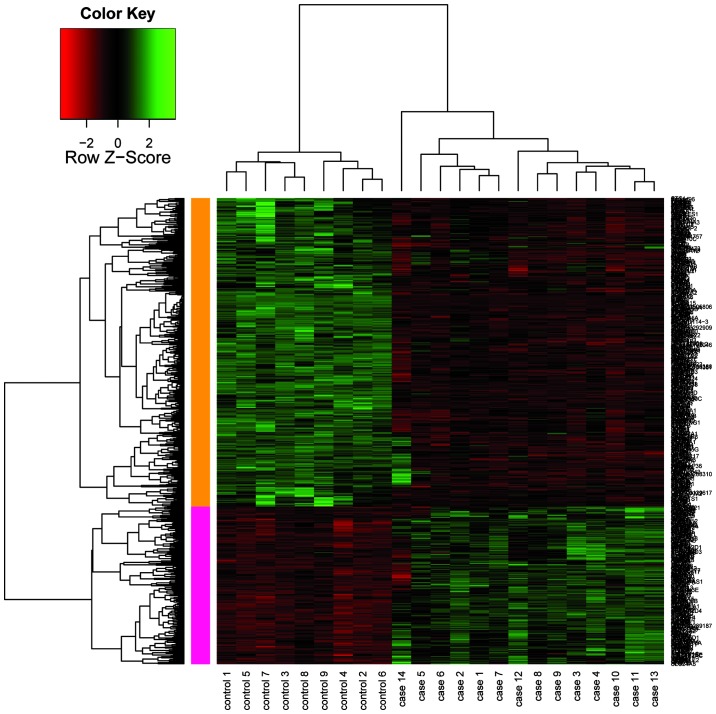
Clustering diagram of DEGs. The bottom abscissa shows names of specimens (control indicates samples of normal pituitary gland, and case indicates samples of pituitary adenoma). The top abscissa indicates clustering of specimens. The right longitudinal axis shows DEGs, while the left longitudinal axis indicates the clustering of DEGs. Red represents downregulated genes and green represents upregulated genes. Two major groups were identified in the clustering diagram of specimens: DEGs and samples of pituitary adenoma. The clustering of DEGs may also be divided into two subgroups: Downregulated and upregulated genes of pituitary adenoma samples. DEG, differentially-expressed genes.

Following the pretreatment of dataset GSE26966 and DEG screening, a total of 624 DEGs, including 211 upregulated genes and 413 downregulated genes, between pituitary adenoma and normal pituitary tissue samples were acquired. A clustering diagram of DEGs is shown in Fig. 1.

### Functional enrichment analysis of DEGs

Significant enrichment GO items of upregulated and downregulated DEGs are shown in Table I. The functions of the downregulated genes in the pituitary adenoma were predominantly associated with the immune response, hormone regulation and cell proliferation. The upregulated genes were associated with cation transport, such as metal ion transport. The stimulation and adjustment of downregulated DEGs on hormones may be associated with hormone secretion in the pituitary gland.

### PPI network construction

**Figure 2:**
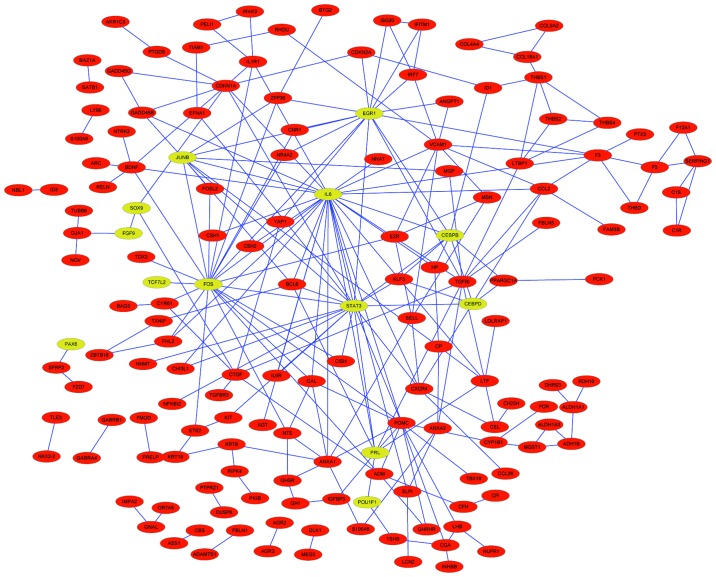
Protein-protein interaction networks of the downregulated differentially-expressed genes in pituitary adenoma. The 145 red nodes indicate the corresponding general proteins of the downregulated genes, while the 13 yellow nodes indicate the corresponding transcription factors.

**Figure 3:**
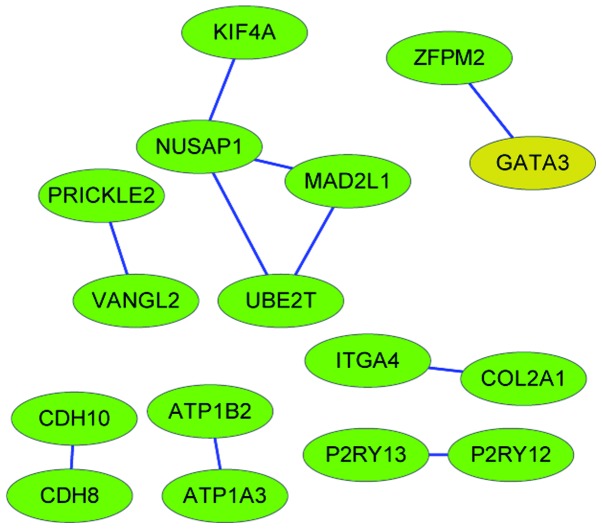
Protein-protein interaction network of the upregulated differentially-expressed genes in pituitary adenoma. The 15 green nodes indicate the corresponding general proteins translated for upregulated genes, while the yellow nodes indicate the corresponding transcription factors.

The PPI network was constructed by PPI pairs based on the STRING database. The PPI network of downregulated genes was composed of 158 nodes and 241 lines (Fig. 2), and the network of upregulated genes consisted of 16 nodes and 10 lines (Fig. 3). In total, 13 transcription factors were included in the PPI network of the downregulated genes, such as early growth response 1 (EGR1), signal transducer and activator of transcription (STAT3), jun B proto-oncogene (JUNB) and FOS, while only the transcription factor GATA-binding protein-3 was included in the PPI network of upregulated genes.

### Functional modules of the PPI network of downregulated DEGs

The PPI network of the downregulated DEGs in pituitary adenoma exhibited centralization, and certain node proteins of the network, including EGR1, STAT3, JUNB and FOS, were the common transcription factors in cancer (23); the PPI network of upregulated DEGs was sparsely populated. Through the comparison between these two PPI networks (Figs. 2 and 3), downregulated genes were observed to be significant in pituitary adenoma. Therefore, the current study focused on the analysis of functional modules in the PPI network of downregulated DEGs.

**Figure 4:**
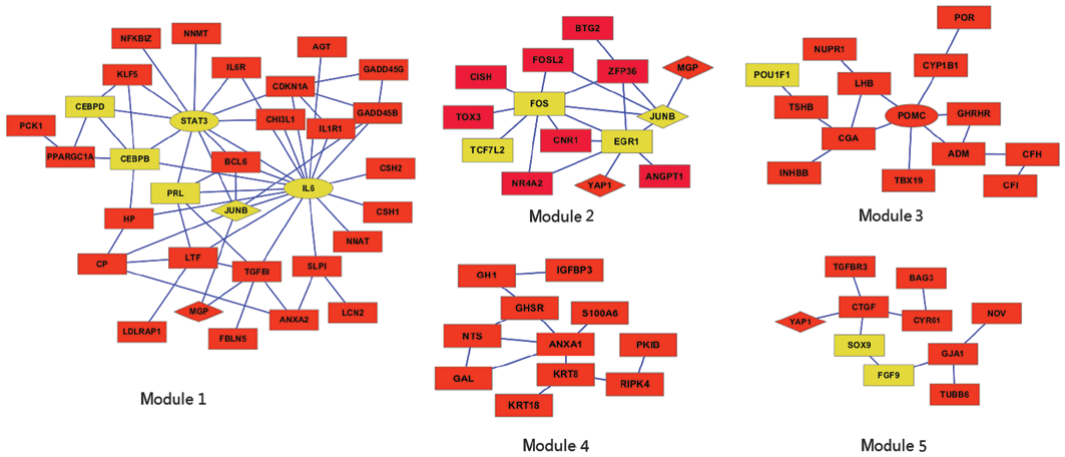
Five modules from the protein-protein interaction networks of the downregulated differentially-expressed genes in pituitary adenoma. The red rectangles represent the general proteins translated by the corresponding downregulated genes in pituitary adenoma; the yellow nodes represent the transcription factors translated by the corresponding downregulated genes in pituitary adenoma. The rhombic nodes represent the proteins existing in several modules at the same time, such as JUNB, MGP and YAP1. Among the five modules, module one exhibited the largest number of nodes, the core nodes of which are the transcription factors downregulated by STAT3 and IL-6.

Five modules were acquired (Fig. 4), with module one exhibiting the largest number of nodes (n=32). In addition to module one, the functions of the remaining four modules were significantly enriched (Table II). In module one, interleukin-6 (IL-6), IL-6-receptor (IL6R) and STAT3 were observed to be enriched in the IL-6-mediated signaling pathway (P<0.05), and IL-6, angiotensinogen, leukemia inhibitory factor, haptoglobin, B-cell lymphoma 6 protein (BCL6), peroxisome proliferator-activated receptor gamma coactivator 1α, low density lipoprotein receptor adaptor protein-1 and STAT3 were enriched in the homeostatic process (P<0.05). Furthermore, the GADD45B and GADD45G were observed to interact with STAT3 and IL-6 in module one. Two types of functions of proteins in module two were identified: The starting of DNA transcription and the response to hormone stimulus. Proteins in modules three and four were predominantly enriched in hormone regulation and metabolism functions.

## Discussion

Following the screening of DEGs from the gene expression profiling of the normal pituitary and pituitary adenoma tissues, differences were identified in the expression of significant genes and proteins associated with pituitary adenoma by functional enrichment analysis and PPI network construction of the DEGs. Furthermore, functional modules of protein interactions were presented with regard to the downregulated protein network. According to these results, the differential expression of genes associated with dysfunction and parasecretion of the pituitary gland, particularly the downregulation of certain transcription factor genes, significantly contributed to the occurrence of pituitary adenoma.

As the results associated with the downregulated genes demonstrated in the four functional modules, a number of proteins interacting as a whole participated in the modulation of the pituitary adenoma, the processes of which were conjoined by key transcription factors. POU1F1 is an important transcription factor found in modules three and four, the function of which is associated with hormone regulation and metabolism. POU1F1 is expressed by the anterior pituitary gland; it is important in body growth and propagation through regulating the expression of PRL, GH, Pit 1 and TSH-β by combining with the promoters described (24). In the current study, a significant decrease of POU1F1 was detected in the pituitary adenoma tissues. This result is supported by a number of studies investigating cases of combined pituitary hormone deficiency. Carlomagno *et al* (25) and Turton *et al* (26) revealed that levels of GH, PRL and TSH decreased in the presence of diverse mutations of the POU1F1 gene. Therefore, we propose that the significantly decreased expression of the POU1F1 gene is a pivotal factor, leading to the reduction of pituitary hormone secretion in pituitary adenoma (27,28). Additionally, it has been reported that two mutation sites of POU1F1 may be associated with the occurrence of combined pituitary hormone deficiency (26).

STAT3 and IL-6, which were found to be the two core nodes in module one, are pivotal transcription factors associated with immune regulation in tumors. Immune abnormalities of a number of tumors have been accompanied by the reduced expression of STAT3 and IL-6 (29). However, no direct correlation has been identified between the occurrence of pituitary adenoma and the expression of the two transcription factors.

The folliculostellate (FS) cells of the normal pituitary gland express IL-6 (30), and the secretion of IL-6 is considered to be independent of human pituitary adenoma subtypes, which was initially determined by Jones *et al* (31) and confirmed by IL-6 mRNA detection (32,33). The production of IL-6 in the normal pituitary gland was hypothesized to be secreted by FS cells (34); however, in pituitary adenomas, the source of IL-6 is the tumor cells (35). High serum IL-6 levels in severe cases of systemic inflammation with bacterial infections (36) indicate that IL-6 increases the cell function with regard to immunity and the inflammation response, as confirmed by experiments with IL-6-deficient mice (37).

STATs are cytoplasmic transcription factors and the key growth factors and mediators of signaling pathways and cytokines, respectively (38,39). Constitutive activation of STAT3 has been observed in prostate, pancreatic, pituitary gland, brain, ovarian and a number of other cancers (40–43). STATs normally exist in combination with the cognate receptor. In response to stimuli, phosphorylation to P-STATs occurs, followed by release from the receptor. P-STATs exert modulations to target gene expression through translocation to the nucleus (29). In the current study, it was demonstrated that the reduced expression of IL6 and STAT3 was associated with pituitary adenoma immune abnormalities. This result was consistent with the conclusion drawn by Kusaba *et al* (44), proposing that P-STAT3 expression is associated with carcinogenesis and tumor invasion in colorectal adenocarcinoma.

As the results in module one demonstrated, the direct combined action and coadjustment between IL-6 and STAT3 was observed on pituitary adenoma development. STAT3 is known to be activated by IL-6, and the deficiency of STAT3 often increases the accumulation of neutrophils (45). Furthermore, IL-6 activity is dependent on the participation of STAT3, which is significant in the transmission of IL-6 signals.

Being predominantly expressed in germinal center (GC) B-cells, BCL6 is also a downregulated protein found in module one. BCL6 is essential in pituitary adenoma due to interactions with several factors, including STAT3, IL-6 and JUNB. A study by Arguni *et al* (46) revealed that activated STATs leads to high BCL6 expression in GC B cells; this supports the findings in the current study, which demonstrated that low expression levels of STATs may decrease BCL6 expression and in turn, contribute to the development of pituitary adenomas.

In conclusion, the current study identified DEGs and proteins between normal pituitary gland and pituitary adenomas through establishing the gene expression profiling and PPI networks. The results of this study indicated that low expression levels of hormonal and immune-related genes facilitates the development of pituitary adenoma. The low expression of IL-6 and STAT3 was important in the dysimmunity of the pituitary adenoma. Furthermore, the low expression of POU1F1 contributed to the reduction in pituitary hormone secretion.

## Figures and Tables

**Table I:** Significant GO terms of DEGs.

DEGs and GO terms	Gene number	Bonferroni adjusted P-value	FDR
Downregulated genes			
GO:0010033 response to organic substance	46	0.000001	0.000001
GO:0009719 response to endogenous stimulus	32	0.000007	0.000005
GO:0042127 regulation of cell proliferation	46	0.000019	0.000015
GO:0009611 response to wounding	36	0.000030	0.000023
GO:0008285 negative regulation of cell proliferation	29	0.000031	0.000024
GO:0009725 response to hormone stimulus	29	0.000045	0.000035
GO:0040008 regulation of growth	26	0.000573	0.000446
GO:0006954 inflammatory response	25	0.000875	0.000681
GO:0048545 response to steroid hormone stimulus	18	0.003978	0.003104
GO:0003006 reproductive developmental process	20	0.018916	0.014869
GO:0006952 defense response	33	0.023125	0.018216
GO:0001501 skeletal system development	22	0.027839	0.021981
GO:0045944 positive regulation of transcription from RNA polymerase II promoter	24	0.028580	0.022575
GO:0002526 acute inflammatory response	12	0.029335	0.023180
GO:0016477 cell migration	20	0.039129	0.031075
GO:0050878 regulation of body fluid levels	14	0.042158	0.033532
GO:0006357 regulation of transcription from RNA polymerase II promoter	36	0.045649	0.036374
GO:0030182 neuron differentiation	26	0.048751	0.038908
Upregulated genes			
GO:0030001 metal ion transport	19	0.001578	0.002293
GO:0006811 ion transport	24	0.004125	0.005999
GO:0006812 cation transport	20	0.004561	0.006635

DEG, differentially-expressed genes; GO, gene ontology; FDR, false discovery rate.

**Table II:** Enriched functions of the five functional modules in the protein-protein interaction network of downregulated differentially-expressed genes.

Module and GO terms	Gene count	Gene list	Fold enrichment	FDR
1				
GO:0070102 interleukin-6-mediated signaling pathway	3	IL6, IL6R, STAT3	450.933	0.02057
GO:0042592 homeostatic process	10	IL6, AGT, LTF, HP, BCL6, CP, PPARGC1A, LDLRAP1, STAT3, PCK1	6.004	0.02706
GO:0045944 positive regulation of transcription from RNA polymerase II promoter	6	EGR1, FOS, NR4A2, YAP1, TCF7L2, JUNB	16.829	0.01404
GO:0009612 response to mechanical stimulus	4	FOS, BTG2, MGP, JUNB	74.330	0.01982
GO:0009719 response to endogenous stimulus	6	FOS, BTG2, NR4A2, MGP, ANGPT1, JUNB	15.417	0.02150
2				
GO:0010033 response to organic substance	7	EGR1, FOS, BTG2, NR4A2, MGP, ANGPT1, JUNB	10.103	0.02166
GO:0006357 regulation of transcription from RNA polymerase II promoter	7	EGR1, FOS, FOSL2, NR4A2, YAP1, TCF7L2, JUNB	10.020	0.02271
GO:0045893 positive regulation of transcription, DNA-dependent	6	EGR1, FOS, NR4A2 YAP1, TCF7L2, JUNB	13.090	0.04738
GO:0042445 hormone metabolic process	5	CGA, CYP1B1, ADM, LHB, GHRHR	45.580	0.00324
3				
GO:0010817 regulation of hormone levels	5	CGA, CYP1B1, ADM, LHB, GHRHR	31.996	0.01323
GO:0035270 endocrine system development	4	CGA, POU1F1, TBX19, GHRHR	56.017	0.04697
GO:0042445 hormone metabolic process	5	CGA, CYP1B1, ADM, LHB, GHRHR	45.580	0.00324
4				
GO:0010817 regulation of hormone levels	5	CGA, CYP1B1, ADM, LHB, GHRHR	31.996	0.01323
GO:0035270 endocrine system development	4	CGA, POU1F1, TBX19 GHRHR	56.017	0.04697

GO, gene ontology; FDR, false discovery rate.
